# Family functioning buffers the consequences of the COVID-19 pandemic for children’s quality of life and loneliness

**DOI:** 10.3389/fpsyg.2022.1079848

**Published:** 2023-01-13

**Authors:** Micah A. Skeens, Kylie Hill, Anna Olsavsky, Jessica E. Ralph, Shivika Udaipuria, Terrah Foster Akard, Cynthia A. Gerhardt

**Affiliations:** ^1^Center for Biobehavioral Health, Nationwide Childrens Hospital, Columbus, OH, United States; ^2^Department of Pediatrics, The Ohio State University School of Medicine, Columbus, OH, United States; ^3^Vanderbilt School of Nursing, Vanderbilt University, Nashville, TN, United States

**Keywords:** COVID-19, pandemic, quality of life, loneliness, family functioning, children

## Abstract

COVID-19 resulted in mass quarantine measures early in the pandemic. This disruption of daily life widened inequities and made children one of the most vulnerable populations during the crisis. This national, cross-sectional “COVID-Kids” study collected data from almost 500 parent–child dyads using standardized measures to better understand the effects of COVID exposure and impact on children’s quality of life and loneliness. Data were collected *via* social media from May to July 2020. According to parent proxy and child self-report, United States children experienced worse quality of life (*p* < 0.0001; *d* = 0.45 and 0.53) and greater child-reported loneliness (*p* < 0.0001) when compared to normative, healthy samples (i.e., children who do not have a chronic medical condition). Older children (*r* = 0.16, *p* = 0.001) and female children (*r* = 0.11, *p* = 0.02) reported greater loneliness. Higher child-reported family functioning scores were associated with better quality of life (*r* = 0.36, *p* < 0.0001) and less loneliness (*r* = −0.49, *p* < 0.0001). Moderated mediation analyses indicated the indirect effect of parent COVID impact on the association between COVID exposure and child quality of life was weaker in the context of better family functioning. Results of this study raise concern for the short-and long-term sequelae of the pandemic on the physical and mental health of children. Healthcare providers and researchers must find new and innovative ways to protect the well-being of children. Strengthening family functioning may buffer the effects of the pandemic and improve overall quality of life in our “COVID Kids.”

## Introduction

In 2020, the novel coronavirus (COVID-19) rapidly spread around the world and was declared a pandemic by the World Health Organization (WHO; Imran et al., 2020). The United States has been one of the most affected countries, with over 96 million infections and almost 1 million deaths as of September 2022 (Team, 2021; Centers for Disease Control and Prevention, 2022). To mitigate the public health crisis, mass quarantine measures (“lockdown”) have included stay-at-home orders, mandated masking, curfews, work from home, and cessation of many in-person activities, including school attendance for children. This disruption of daily life and routines widened inequities and made children and adolescents one of the most vulnerable populations during the crisis (Quinn et al., 2016; Golberstein et al., 2020; Wang et al., 2020).

While much is known about children’s reactions to natural disasters (Pina et al., 2008; Schoenbaum et al., 2009; McDermott and Cobham, 2012), less is known about their response to pandemics. Exposure to natural disasters can result in symptoms of post-traumatic stress, depression, anxiety, and behavioral problems in children (Pina et al., 2008; Schoenbaum et al., 2009). Among the few studies that have examined past pandemics’ impact on children’s mental health (i.e., equine influenza, H1N1, and Ebola), negative psychological outcomes such as stress, helplessness, and risky behavioral problems have been reported (Meherali et al., 2021). Although the direct physical sequelae of the COVID-19 virus may be less severe in children, the pandemic has resulted in significant indirect effects on children’s physical, social, and mental health (Ghosh et al., 2020; Golberstein et al., 2020; Wang et al., 2020; Zar et al., 2020; Nobari et al., 2021; Samji et al., 2021; Elharake et al., 2022). In one of the earliest surveys of over 2,000 youth with mental health needs in the United Kingdom, over 80% reported the pandemic resulted in worse mental health, 87% reported greater social isolation, and 31% had reduced access to mental healthcare (YoungMinds, 2020). Among 115 adolescent girls in the Netherlands, one in four reported depressive symptoms above the clinical cut-off during the first COVID-19 lockdown, and had an increased risk of depressive symptoms when they reported poor family functioning (Vacaru et al., 2022). In another study, children in both Canada and China had drastically reduced rates of physical activity and increased sedentary behavior during the pandemic (Moore et al., 2020; Xiang et al., 2020). Chinese children had increased distraction, irritability, and fear in the early months of quarantine (Jiao et al., 2020).

In the United States, similar effects of the COVID-19 pandemic on children’s quality of life and psychosocial functioning have been reported. Longitudinal studies of youth well-being during the pandemic found that children and adolescents reported higher levels of internalizing and externalizing problems when they experienced more pandemic-related stressors (Rosen et al., 2021; Weissman et al., 2021). Children in the United States are also engaging in less physical activity and greater amounts of screen time, despite findings that suggest better health behaviors are associated with improved mental health outcomes (Tandon et al., 2021). Stress and anxiety in parents, as well as exposure to increased information regarding the pandemic, may cause uncertainty, fear, and other psychological and social consequences for children (Fiorillo and Gorwood, 2020). A study of a community-based sample of mothers and children found that pandemic-related stressors led to an increase in maternal mental health symptoms, which then predicted greater psychopathology symptoms among adolescents (Lengua et al., 2022). Moreover, COVID-19-related threat information from parents and the community contributed to greater fear in children, particularly for younger children (Uy et al., 2022). While parent distress has been shown to contribute to child stress and post-traumatic stress disorder during disaster (Kelley et al., 2010), family, social, and school connectedness may be protective for high risk youth (Foster et al., 2017) and improve long-term adult health (Steiner et al., 2019). A greater understanding of the role of connectedness, particularly its potential to buffer the effects of the pandemic, is needed.

The current pandemic provides a real-world opportunity for investigation of the effects of severe stress on social and emotional well-being in children while experiencing disruption of daily routines and social isolation. A better understanding of this experience provides an opportunity for healthcare providers, educators, and caregivers to identify high risk youth and engage in a rapid response or prevent future sequelae. Thus, our objective was to examine quality of life and loneliness among United States children in the early months of the COVID-19 pandemic and to explore demographic and family factors related to child well-being. Specifically, we examined the mediating role of COVID-19 impact and the moderating roles of family functioning and communication with friends on associations between COVID-19 exposure and child quality of life and loneliness. We hypothesized that greater reported COVID-19 impact on parents or children would directly and significantly affect the quality of life of children.

## Materials and methods

### Participants

This paper presents a cross-sectional analysis of quality of life and loneliness among United States children early in the COVID-19 pandemic. Eligible parents were older than 18 years of age and had a child enrolled in public school prior to the COVID-19 pandemic. Youth were: (a) 8–17 years of age, (b) English speaking, (c) typically enrolled in school outside of the home, and (d) living with a participating caregiver. Children with developmental delays and children who were home schooled prior to the COVID-19 pandemic were excluded.

### Ethical considerations

Institutional review board (IRB) approval was obtained (STUDY00001019). The institutional IRB determined informed written consent would not be required, as participants were providing implied consent by clicking on the Facebook Ad and completing all questions. The Facebook Ad led all participants to a study summary description. Participants were instructed by proceeding to complete the study questions, they were providing consent to participate. No protected health information was collected, the study was anonymous, and participants could stop participation at any time. No questions were required to be answered to proceed to other questions.

### Data collection

Data on the effects of COVID-19 on school-aged children were collected from parent–child dyads through a pay per click ad campaign on Facebook. Consent and assent were implied *via* participants’ voluntary completion of the anonymous survey, which they could exit out of at any time. Parents completed a survey about the effects of the COVID-19 pandemic on school-aged children. After parents completed their portions, the survey was then directed to the oldest child who was willing to complete child measures. Data collection ended after an 8-week period.

#### Internet based recruitment

To adapt to the challenges of conducting clinical research during the pandemic and quickly obtain a reasonable sample size, families were recruited remotely using an 8-week pay per click Facebook Ad campaign from May 2020 until July 2020. Because Facebook users must be at least 13 years of age, the ad invited parents to participate in a survey about the effects of the COVID-19 pandemic on school-aged children with a chance to win a $100 Amazon gift card. After clicking on the Facebook link, a summary describing the purpose, benefits, risks, time commitment, and rights as a research volunteer was provided to all parents, this included an invitation to their child to participate.

#### Measures

##### Demographic characteristics

Data were collected from the parent about themselves and partner (if applicable), including number of children, sex, race, ethnicity, marital status, geographic location, income, employment status, occupation, and COVID-19 exposure. Parents were also asked about the participating child’s age, grade, sex, race, ethnicity, receipt of home instruction, method of home instruction, social contact with friends, and method of communication with friends.

##### COVID-19 exposure and family impact scale

This measure was created using a rapid iterative process by members of the Center for Pediatric Traumatic Stress (Kazak et al., 2021). It captures exposure to potentially traumatic aspects of COVID-19 and assesses the impact of the pandemic on the family. Part 1 consists of 28 yes/no responses measuring exposure to COVID-19 and associated disruptions, generating a total exposure score. Sample items include having a stay-at-home order, school closure, or a family member continue to work outside the home. Internal consistency for COVID-19 exposure in our sample was *α* = 0.63. Part 2 is comprised of 12 items measuring the impact of the COVID-19 pandemic. Ten items are rated on a five-point scale (i.e., impact on parenting, independence, how family members get along), and two items assess distress on a 10-point scale. Higher summary scores indicate greater exposure to COVID-19 stress and more negative impact/distress. Internal consistency for COVID-19 impact in our sample was *α* = 0.83. Because a child version of the CEFIS was unavailable, we adapted seven questions from the adult version to measure impact. Internal consistency for child-reported COVID-19 impact in our sample was *α* = 0.75.

##### Pediatric Quality of Life Inventory™ 4.0 generic core scales

Children completed this 23-item measure, which includes four scales: Physical Functioning, Emotional Functioning, Social Functioning, and School Functioning (Varni et al., 2003). Items are rated from 0 to 4 and reverse scored. A total score is calculated ranging from 0 to 100, with higher scores indicating better quality of life. Reliability and validity have been documented, and data allow for comparisons to means from healthy populations (i.e., children ages 2–16 who do not have a chronic medical condition; Varni et al., 2003; Huang et al., 2013). Additionally, internal consistency for COVID-19 exposure in our sample was *α* = 0.91. Children are considered to have compromised quality of life if scores fall more than one standard deviation below the population mean of 82.87 for total functioning.

##### NIH toolbox and PROMIS measures

Children completed the NIH Toolbox Loneliness (seven items) and PROMIS® short form Family Relationship (four items) measures. Items are rated using a 5-point scale, with higher scores indicative of higher levels of the construct, which can be positive (family functioning) or negative (loneliness). Both measures generate T-scores (*M* = 50; *SD* = 10) normed to the general population. The NIH Toolbox and PROMIS® are well-validated and reliable measures of self-reported health outcomes (Bevans et al., 2010). Internal consistency for loneliness in our sample was *α* = 0.92 and for family functioning was *α* = 0.87.

#### Statistical analysis

Data were analyzed using SPSS software, version 26. Descriptive statistics were calculated for demographic characteristics and primary variables of interest. Parent and child reported PedsQL scores were compared to the mean PedsQL score for the healthy population (Varni et al., 2003) using *t*-tests (*α* = 0.05; two-way). Child reported loneliness was analyzed similarly. To identify potential covariates prior to running multivariate models, Pearson correlations (*α* = 0.05; two-way) were conducted between primary dependent variables (i.e., quality of life, loneliness) and demographic factors, as well as COVID exposure, COVID impact, family functioning, and communication with friends.

The PROCESS macro for SPSS (Hayes, 2017) was used to conduct moderated mediation analysis using ordinary least squares regression in four separate models. Child age, sex, ethnicity and prior family income was controlled for in each model where COVID-19 exposure was the primary independent variable, COVID-19 impact was the mediator, and either the PEDSQL or loneliness was the outcome; then communication with friends or family functioning were tested as potential moderators of the association between COVID impact and quality of life or loneliness. Indirect effects were assessed using 95% bias-corrected confidence intervals based on 10,000 bootstrap samples; the effect was considered significant when confidence intervals did not contain zero. Thereafter, conditional indirect effects of exposure on outcome was examined through the mediator, followed by second stage moderation. A simple mediation analysis was conducted when the moderator was non-significant.

## Results

### Sample characteristics

**Figure 1:**
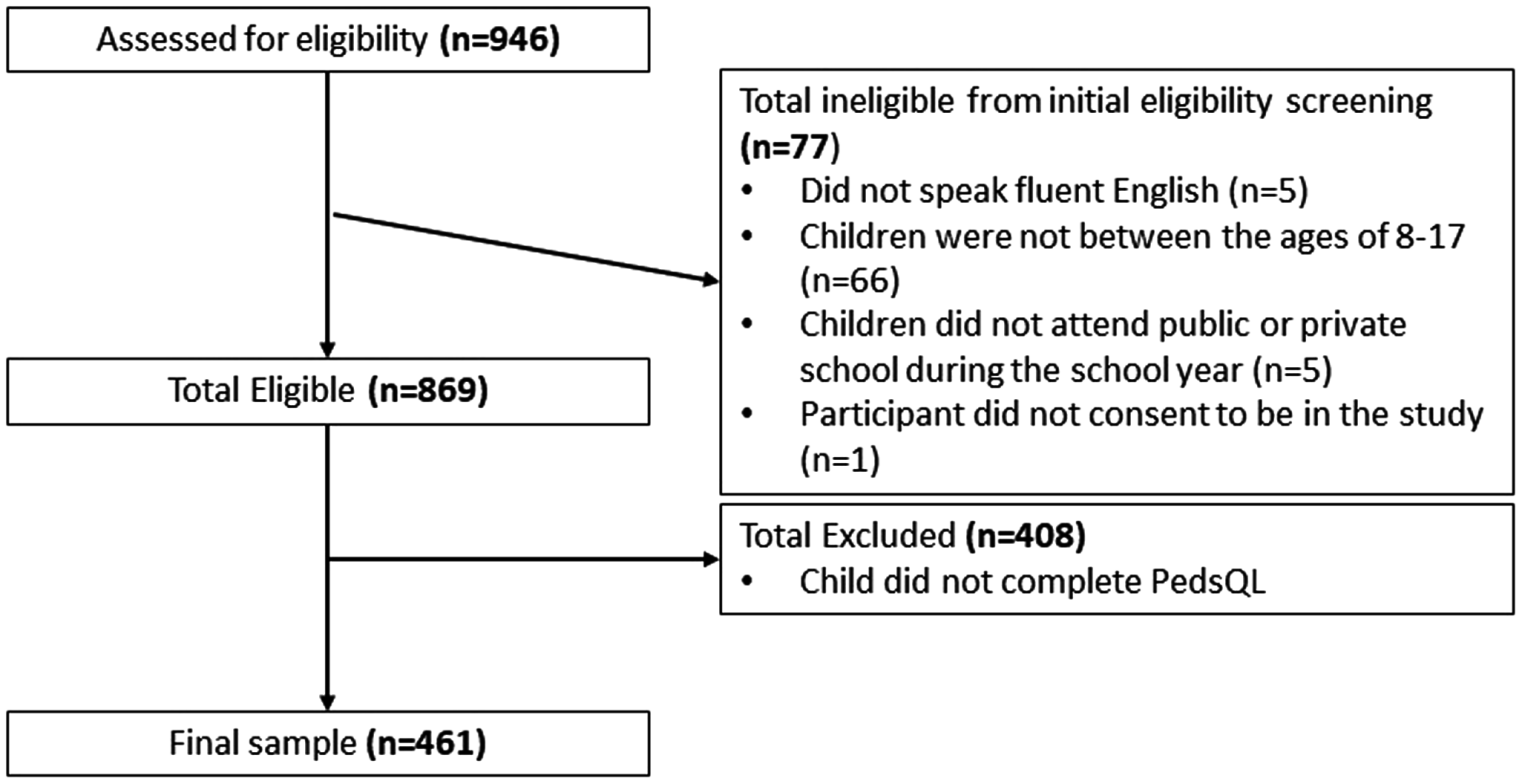
Consort diagram describing sample included.

A total of 461 parent/child dyads (Figure 1 consort diagram) completed self-report measures. Tables 1, 2 provide demographic descriptions of parent and child participants. Child participants were equally distributed by sex (49% female, *n* = 223), with a mean age of 11.8 years old (range 8–17, *SD* = 2.72). Most parent participants were mothers (95% female, *n* = 435), White (91%, *n* = 418), and non-Hispanic (93%, *n* = 423). While 26% (*n* = 118) of parents were unemployed, almost half reported an annual income prior to COVID-19 greater than $100,000. Most families were from the Midwest; however, all 50 U.S. states were represented.

**Table 1:** Child dyad demographic characteristics (*N* = 461).

	Mean (SD)* or *n* (%)
Child age in years (SD)	11.85 (2.72)
Gender	
Male	236 (51.4%)
Female	223 (48.6%)
Race	
American Indian/Native American	4 (0.9%)
Asian	19 (4.1%)
Black or African American	25 (5.4%)
Native Hawaiian/Pacific Islander	3 (0.7%)
White	416 (90.2%)
Other (fill-in text response)	16 (3.5%)
Ethnicity	
Hispanic or Latino	43 (9.4%)
Non-Hispanic	415 (90.6%)

*SD, standard deviation.

**Table 2:** Parent dyad demographic characteristics (*N* = 461).

	Mean (SD)* or *n* (%)
Gender	
Male	23 (5.0%)
Female	435 (95.0%)
Race	
American Indian/Native American	4 (0.9%)
Asian	14 (3.0%)
Black or African American	14 (3.0%)
Native Hawaiian/Pacific Islander	3 (0.7%)
White	418 (90.7%)
Other (fill-in text response)	16 (3.5%)
Ethnicity	
Hispanic or Latino	33 (7.2%)
Non-Hispanic	423 (92.8%)
Income	
Under $25,000	31 (6.8%)
$25,001–$50,000	57 (12.6%)
$25,001–$75,000	54 (11.9%)
$75,001–$100,000	86 (18.9%)
$100,001–$150,000	137 (30.2%)
More than $150,000	85 (18.7%)
Other	4 (0.9%)
Average years of education (SD)	15.53 (4.57)
Current employment status	
Working full-time (>30 h/week)	264 (57.6%)
Working part-time (<30 h/week)	76 (16.6%)
Unemployed	118 (25.8%)
Current relationship status	
Single (includes separated, divorced, and widowed)	59 (12.8%)
Married or living with Someone	400 (87.2%)

*SD, standard deviation.

### COVID exposure and impact

#### Parent report

The mean COVID-19 exposure score was 8.08 (*SD* = 2.59) on a scale of 25. The largest proportion of COVID-19 exposure reflected indirect events, such as closure of schools and daycares (99.8%, *n* = 460), stay at home orders (96.5%, *n* = 445), disruption in education (94.4%, *n* = 435), missing important events (85.9%, *n* = 396), and inability to visit/care for family members (78.7%, *n* = 362). Only 12.2% (*n* = 56) of participants had direct exposure due to a family members’ diagnosis of COVID-19, and COVID-related deaths affected only 1.1% (*n* = 5) of the sample.

The average COVID impact score was 35.03 (*SD* = 6.91) on a scale of 0 to 50. Parents reported a mean distress score of 6.20 (*SD* = 2.11) for themselves and 5.97 (*SD* = 2.26) for children. Children’s self-reported distress score was 5.04 (*SD* = 2.49), which was significantly lower than parent report of child distress *t*(457) = −9.13, *p* < 0.0001. However, child-reported parent distress scores were similar to parent self-report 6.15 (*SD* = 2.53). Overall, both parents and children reported higher distress scores for parents than children.

#### Child report

Because a child version of the CEFIS was unavailable, we adapted seven questions from the adult version to measure impact. Approximately 30% (*n* = 135) of children reported COVID-19 improved how family members got along, while 38% (*n* = 174) reported it made it worse. About 44% (*n* = 172) reported a negative impact on sibling relationships. Most children reported negative effects on emotional well-being, including worry (58.5%, *n* = 266) and mood (56.3%, *n* = 255).

### Quality of life and loneliness

Comparisons to normative, healthy population (i.e., healthy children) means for parent and child reported quality of life and child reported loneliness scores are displayed in Table 3. Both parent and child-reported PedsQL total scores were significantly lower (worse) than the normative mean (*p* < 0.0001; *d* = 0.45 and 0.53; Varni et al., 2003). Parent and child-reported domains of emotional, physical, and school functioning were also significantly below the normative mean (*p* < 0.0001); however social functioning was not. Dyad reports of child quality of life were strongly correlated (*r = 0*.75; *p* < 0.0001). The mean child-reported loneliness score was 56.12 (*SD* = 11.27), which was significantly higher (worse) than normative scores.

**Table 3:** Comparison of parent and child reports of child quality of life to norms.

	Normative sample *M* (*SD*)	Current sample *M* (*SD*)	df*	*t*-value	Pr > |*t*|**	(95% CI)***
PEDSQL child report						
Total functioning	82.87 (13.16)	75.35 (15.12)	6,431	11.69	**<0.0001**	6.26–8.78
Physical functioning	86.86 (13.88)	80.67 (18.47)	6,421	8.98	**<0.0001**	4.84–7.54
Emotional functioning	78.21 (18.64)	63.41 (21.18)	6,420	16.26	**<0.0001**	13.01–16.59
Social functioning	84.04 (17.43)	82.20 (17.77)	6,407	2.18	**0.03**	0.18–3.50
School functioning	79.92 (16.93)	71.91 (18.57)	6,367	9.71	**<0.0001**	6.39–9.63
PEDSQL parent report						
Total functioning	81.34 (15.92)	74.12 (16.16)	10,319	7.09	**<0.0001**	5.22–9.22
Physical functioning	83.26 (19.98)	78.00 (20.15)	10,300	4.13	**<0.0001**	2.76–7.76
Emotional functioning	80.28 (16.99)	59.83 (21.45)	10,294	18.74	**<0.0001**	18.31–22.59
Social functioning	82.15 (20.08)	82.72 (17.49)	10,285	0.45	0.66	−3.08–1.94
School functioning	76.91 (20.16)	73.93 (20.79)	8,715	16.09	**<0.0001**	18.25–23.33
Loneliness score	50.00 (10.00)	56.12 (11.27)	460	11.66	**<0.0001**	5.09–7.15

* df = Degrees of freedom.

** Pr > |*t*| = The *p*-value of a *t*-test.

*** 95% CI = 95% Confidence Interval.

Bold values are statistically significant.

### Factors associated with child quality of life and loneliness

Table 4 includes correlations between demographic factors, child quality of life, and child loneliness. Older children reported greater loneliness (*r* = 0.16, *p* = 0.001). Females also had greater loneliness than males (*r* = 0.11, *p* = 0.02). Higher prior income was significantly correlated with better child-reported overall quality of life (*r* = 0.21, *p* < 0.0001). Higher child-reported family functioning scores were strongly associated with better quality of life (*r* = 0.36, *p* < 0.0001) and less loneliness (*r* = −0.49, *p* < 0.0001). Child communication with friends was also significantly correlated with quality of life (*r* = 0.19, *p* < 0.0001) and loneliness (*r* = −0.13, *p* = 0.006).

**Table 4:** Correlations between demographic characteristics and child report of quality of life and loneliness.

Variable									
1. PEDSQL total functioning									
2. Loneliness	**−0.60** **								
3. Family relationships	**0.36** **	**−0.49** **							
4. Communication with friends	**0.19** **	**−0.13** **	−0.01						
5. Child age	−0.07	**0.16** **	**−0.15** **	**0.21** **					
6. Child sex	0.03	**0.11** *	−0.03	**0.15** **	−0.01				
7. Child ethnicity	**0.13** **	0.03	−0.01	0.08	−0.02	−0.06			
8. Prior income	**0.21** **	−0.01	**−0.12** *	**0.13** **	0.03	−0.06	**0.17** **		
9. COVID exposure	**−0.20** **	0.06	−0.05	**−0.09** *	−0.06	0.02	**−0.16** **	**−0.26** **	
10. COVID impact	**−0.30** **	**0.25** **	**−0.18** **	−0.09	−0.06	−0.05	**0.11** *	−0.06	**0.14** **

* *p* < 0.05.

** *p* < 0.01.

Bold values are statistically significant.

### Exploration of communication with friends and family functioning as moderators of indirect effects

**Figure 2:**
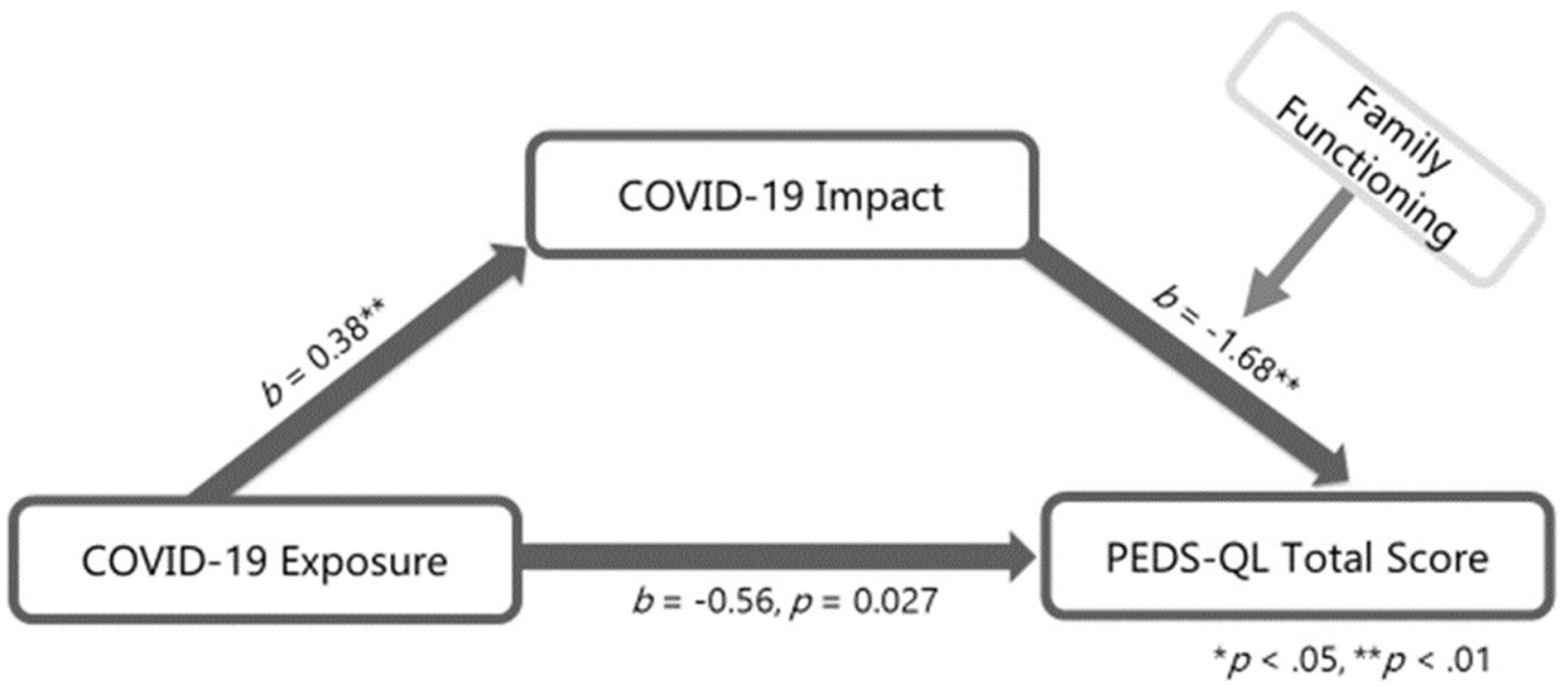
Quality of life moderated mediation. Interaction between COVID-19 Impact and family functioning *b* = 0.03, *p* = 0.02; *r*-squared for the model: 0.26; index of moderated mediation = 0.01, 95% CI [0.001, 0.02]; this model controls for age, gender, ethnicity and prior family of which income and ethnicity was significantly associated with PEDS-QL total score.

**Figure 3:**
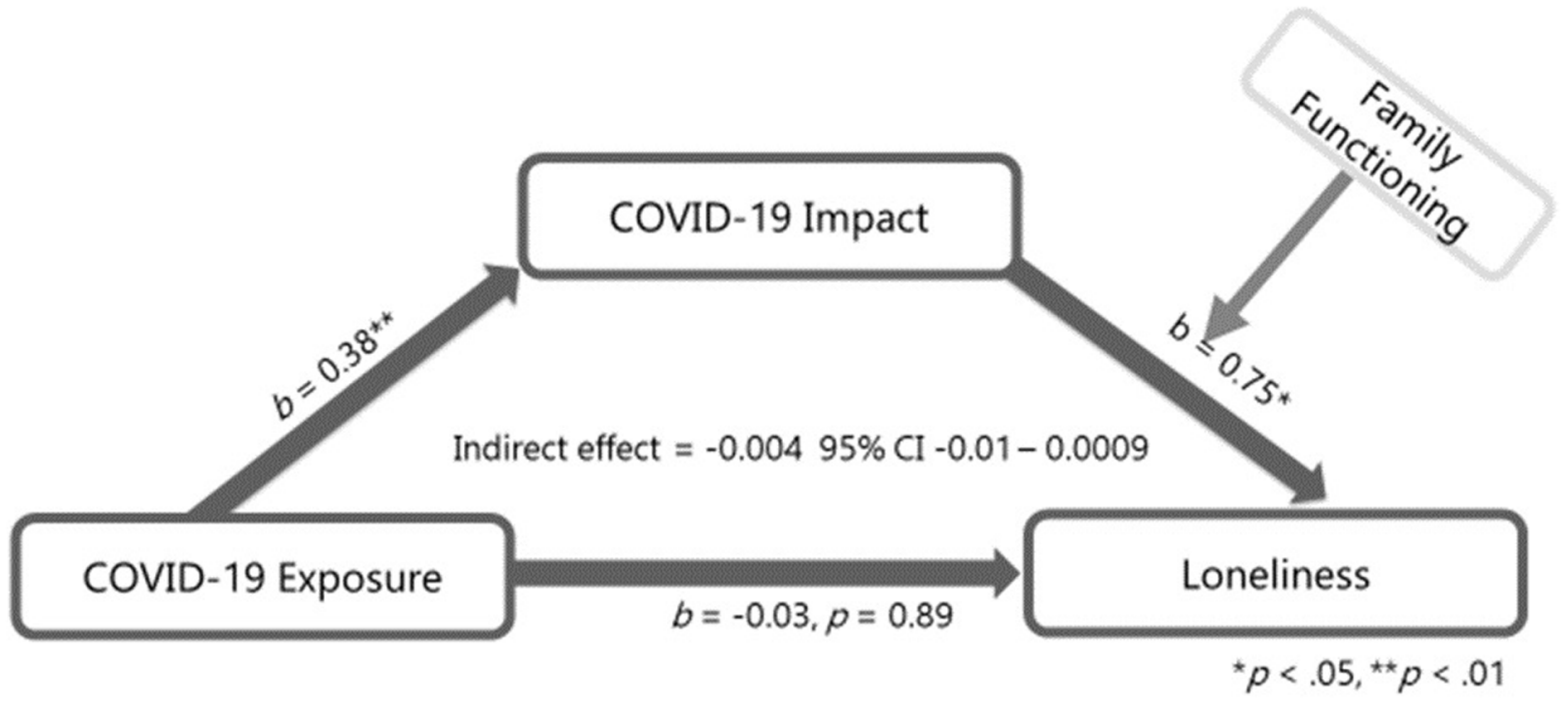
Loneliness moderated mediation. Interaction between COVID-19 Impact and family functioning *b* = −0.01, *p* = 0.19; *r*-squared for the model: 0.30; this model controls for age, gender, ethnicity and prior family income of which gender and age were significantly associated with PEDS-QL total score.

**Figure 4:**
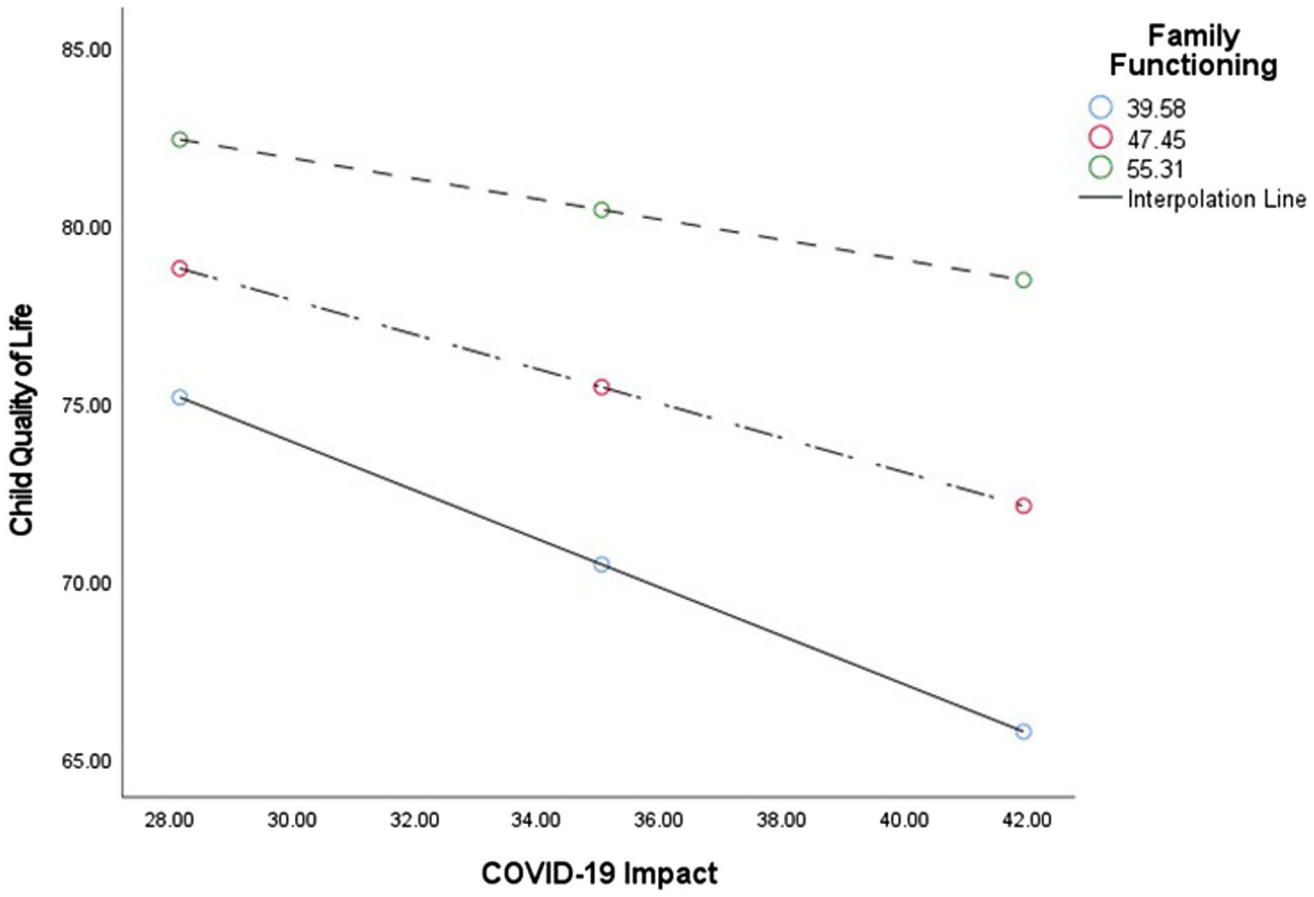
Simple slopes. Illustration of the simple-slope analyses for Family functioning with child quality of life and COVID-19 impact scores.

**Figure 5:**
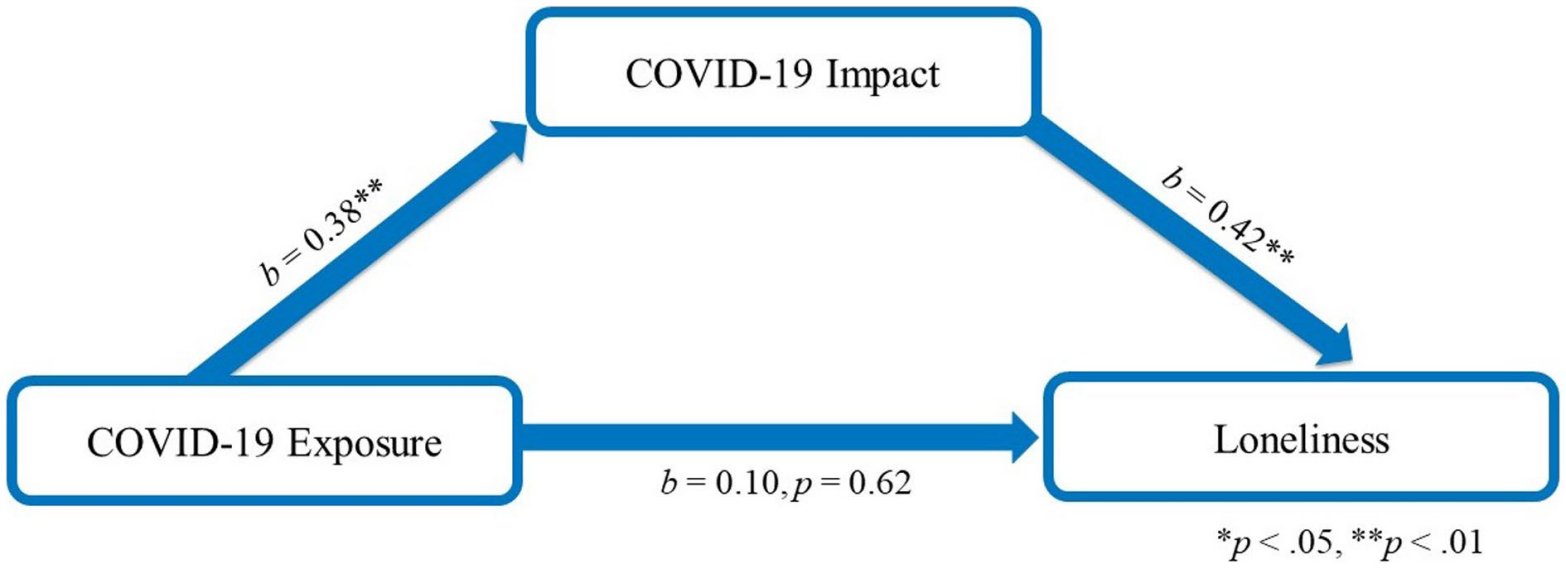
Loneliness mediation model. R-squared for the model: 0.10; this model controls for age, gender, ethnicity and prior family income of which gender and age were significantly associated with loneliness total score.

We expected that the indirect effect of parent COVID-19 impact on associations between COVID-19 exposure and both child quality of life and loneliness would vary in the context of communication with friends and family functioning. Moderated mediation analyses indicated that communication with friends did not affect the strength of these associations (95% CI: −0.09 to 0.34 for quality of life and −0.24 to 0.08 for loneliness); however, the indirect effect of parent COVID impact was weaker in the context of better family functioning for quality of life (95% CI: 0.001 to 0.024), but not for loneliness (95% CI: −0.01 to 0.0009; Figures 2, 3; Simple Slopes in Figure 4). Given that our moderators did not have an effect on our moderated mediation loneliness models, a simple mediation analysis was conducted and was significant (95% CI: 0.04 to 0.31; Figure 5).

## Discussion

While the direct physical effects of COVID-19 on children appear less severe than adults (Ghosh et al., 2020; Zar et al., 2020) results from this study early in the pandemic suggest United States children experienced worse quality of life and greater loneliness when compared to normative samples. These outcomes were worse for girls and older children and raise concern for short-and potentially long-term mental health sequelae due to the pandemic. The effects of the pandemic on children were partially explained by COVID-related distress in parents. While social connection with friends did not buffer this indirect effect, better family functioning appeared to be protective. These findings are particularly relevant for healthcare providers, educators, and caregivers, who may be in a position to assist children in the midst of the pandemic.

Children in our study also experienced greater loneliness than normative samples, which has been described in adults (Van Tilburg et al., 2020; Wickens et al., 2021), but less so in youth during the pandemic. Although an anticipated result of public health restrictions during COVID-19, loneliness is not benign. A recent systematic review found an association between loneliness and mental health problems, specifically depression and anxiety in children and adolescents (Loades et al., 2020). Notably, loneliness was also associated with mental health problems up to 9 years later (Loades et al., 2020). It is also connected with increased depression in girls and social anxiety in boys (Mak et al., 2018; Liu H. et al., 2020). This is concerning given prolonged social distancing and the length of time adolescents have been remote learning during the COVID-19 pandemic.

Drastic changes to lifestyle, school, and physical activity have psychosocial consequences for children (Wang et al., 2020). During the pandemic, home confinement, social distancing from peers and extended family, fear of infection for self or family, and lack of educational resources create feelings of uncertainty and anxiety in children (Imran et al., 2020), which can lead to more serious effects on mental health. In our study, both parents and children reported lower overall quality of life for children when compared to normative samples. All domains of quality of life (total, physical, emotional, and school) except the social domain were affected. This finding is interesting given most children were not physically attending school and social distancing measures were in place to mitigate spread of the virus. This decrease in health-related quality of life has been reported among children in other countries as well (Xie et al., 2020; Ravens-Sieberer et al., 2021).

Our findings indicated worse quality of life for adolescents (i.e., 12–18) relative to children (i.e., 8–12), as well as girls relative to boys. This is similar to the Canadian study that also reported high depression and loneliness in adolescent girls (Castellino et al., 2012) and a recent meta-analysis reporting an increased prevalence of clinically elevated anxiety in females (Racine et al., 2021). Additionally, a United Kingdom study on mental health and loneliness in adolescents during the COVID-19 pandemic reported a significant association between loneliness and being female (Cooper et al., 2021). These results are not surprising given the influence of peer contact on well-being as well as the importance of developing independence in adolescence (Fegert et al., 2020). Adolescents, specifically adolescent girls, are a population vulnerable to mental health concerns, such as depression, in the healthiest of times (Ellis et al., 2020). The COVID-19 pandemic brought about distanced or virtual peer relationships, isolation from friends, restriction in extracurricular activities, and missed major life events. These factors are likely to accentuate risks and require special attention.

Data suggest parental stress influences outcomes in children (Schor, 2003; Louie et al., 2017; Ren et al., 2020; Spinelli et al., 2020; Calvano et al., 2022). One study during the pandemic found parental presence may decrease child stress (Wang et al., 2020). Similarly in our study, when controlling for age, sex, and income, parent reported COVID-impact (distress) was a contributing factor to worse overall quality of life and loneliness reported by children. This could be expected when one considers the stress the pandemic has elicited in parents. In a study examining caregiver strain among parents (a majority of whom were mothers) over 75% of parents reported the strain of caregiving as moderate or high during the COVID-19 pandemic (Radomski et al., 2022). Parents have experienced uncertainty related to physical health and financial strain due to unemployment and decreased wages (Achdut and Refaeli, 2020; Sharma et al., 2020). Parents have been faced with additional pressures of working from home, blurring boundaries between work and family (Cusinato et al., 2020), while balancing remote learning and lack of access to childcare or extended family caregivers. In addition, studies have shown that social isolation can negatively affect adult psychological health (Liu J. J. et al., 2020), further adding to parental stress during the pandemic.

We expected that the ability to maintain social connections and family functioning would be protective for children’s well-being. However, communication with friends, direct or virtually, did not attenuate the association between parent-reported COVID impact and quality of life or loneliness in children. Other studies have also shown worsening depression with increased virtual communication with friends, but no association with loneliness (Ellis et al., 2020). However, family functioning was protective for children, which is similar to findings suggesting less depression or depressive symptoms in adolescents that reported increased family time or experienced higher quality of family functioning (Ellis et al., 2020; Vacaru et al., 2022). Another study of 93 parent–child dyads reported that better family functioning was positively associated with higher health-related quality of life in children (Taha et al., 2022). Furthermore, developmental literature supports the protective role of positive family relationships for children exposed to adversity (Crum and Moreland, 2017; Masten, 2018). Thus, in our study, children were more effected by parental COVID-19 impact and strong family relationships were able to buffer the reported decrease in quality of life and increase in loneliness. Despite the perception adolescents prioritize friends (Laursen and Veenstra, 2021), our findings emphasize the need for strengthening family relationships during times of crisis.

To our knowledge, this is one of few studies using a large sample of parent–child dyads to examine protective factors related to quality of life and loneliness during the pandemic. However, findings should be considered in the context of several limitations. The use of social media to recruit participants is not without criticisms, as it could introduce ascertainment bias and restricted participation of anyone without social media access. We also asked for participation from the oldest willing child, further limiting generalizability. Parents were primarily White, non-Hispanic mothers. While considerable efforts were made to increase diversity, future research with under-represented populations is needed. The overall COVID exposure score in this sample was low, which may reflect the early timing of data collection. In addition, examination of effect sizes indicated some associations, although significant, were relatively weak. Identification of other robust factors associated with child well-being is important. Given the cross-sectional nature of this study, further investigation into the long-term effects of the COVID-19 pandemic on quality of life and emotional well-being is needed.

Despite limitations, this study increased our understanding of the collateral damage occurring in a large sample of United States children during the COVID-19 pandemic. While they may largely escape the direct physical consequences, most children will suffer from some decline in their quality of life and/or emotional well-being. These findings also reinforce the idea that the family unit and parent distress are important contributing factors to child outcomes during the pandemic. Pediatricians, teachers, and community members should assist parents in recognizing the contribution of stress on child outcomes, and strong efforts must be aimed at mitigating this stress and encouraging parental self-care. We can only hope that the resilience that often sustains children’s development will allow them to recover from the repercussions of the pandemic. In the meantime, we must do everything possible to provide them opportunities for a normal childhood and improve their long-term physical and emotional well-being.

## Conclusion

Children are a vulnerable population deeply affected by the unintended consequences of the pandemic. Although the peak of the initial pandemic may have passed, a shift to mitigating the harm invoked by the pandemic on child well-being is necessary. Healthcare providers and researchers alike must find new and innovative ways to protect child mental health, strengthen family functioning, and thus improve overall long-term quality of life of our “COVID Kids.”

## Data availability statement

The raw data supporting the conclusions of this article will be made available by the authors, without undue reservation.

## Ethics statement

The studies involving human participants were reviewed and approved by Nationwide Children’s Hospital Institutional Review Board. Written informed consent from the participants’ legal guardian/next of kin was not required to participate in this study in accordance with the national legislation and the institutional requirements.

## Author contributions

MS and CG developed the concept and design of the study. MS, KH, and AO were responsible for conducting the study. MS, AO, KH, JR, and SU analyzed the data and drafted this manuscript. CG, TA, and JR provided revisions. All authors contributed to the article and approved the submitted version.

## Funding

The authors declare that this study received funding from a Nationwide Children’s Intramural Grant.

## Conflict of interest

The authors declare that the research was conducted in the absence of any commercial or financial relationships that could be construed as a potential conflict of interest.

## Publisher’s note

All claims expressed in this article are solely those of the authors and do not necessarily represent those of their affiliated organizations, or those of the publisher, the editors and the reviewers. Any product that may be evaluated in this article, or claim that may be made by its manufacturer, is not guaranteed or endorsed by the publisher.

## Abbreviations

CEFIS: COVID-19 Exposure and Family Impact Scale
PedsQL: Pediatric Quality of Life Inventory
